# Surgical treatment for recurrent cholangiocarcinoma: a single-center series

**DOI:** 10.3389/fonc.2023.1169133

**Published:** 2023-04-18

**Authors:** Laura Fernández, Mikel Gastaca, Eva Alonso, Mikel Prieto, Patricia Ruiz, Alberto Ventoso, Ibone Palomares, Arkaitz Perfecto, Andrés Valdivieso

**Affiliations:** ^1^General Surgery Department, Hospital de Urduliz, Urduliz, Spain; ^2^Hepatobiliary Surgery and Liver Transplantation Unit, Biocruces Bizkaia Health Research Institute, Hospital Universitario Cruces, Bilbao, Spain; ^3^Facultad de Medicina y Odontología, Universidad del País Vasco/ Euskal Herriko Unibertsitatea (UPV/EHU), Leioa, Spain; ^4^General Surgery Department, Hospital Universitario Cruces, Bilbao, Spain

**Keywords:** cholangiocarcinoma, recurrence, surgical treatment, outcomes, chemotherapy

## Abstract

**Purpose:**

The present study aims to assess the results obtained after surgical treatment of cholangiocarcinoma (CC) recurrences.

**Methods:**

We carried out a single-center retrospective study, including all patients with recurrence of CC. The primary outcome was patient survival after surgical treatment compared with chemotherapy or best supportive care. A multivariate analysis of variables affecting mortality after CC recurrence was performed.

**Results:**

Eighteen patients were indicated surgery to treat CC recurrence. Severe postoperative complication rate was 27.8% with a 30-day mortality rate of 16.7%. Median survival after surgery was 15 months (range 0-50) with 1- and 3-year patient survival rates of 55.6% and 16.6%, respectively. Patient survival after surgery or CHT alone, was significantly better than receiving supportive care (p< 0.001). We found no significant difference in survival when comparing CHT alone and surgical treatment (p=0.113). Time to recurrence of <1 year, adjuvant CHT after resection of the primary tumor and undergoing surgery or CHT alone versus best supportive care were independent factors affecting mortality after CC recurrence in the multivariate analysis.

**Conclusion:**

Surgery or CHT alone improved patient survival after CC recurrence compared to best supportive care. Surgical treatment did not improve patient survival compared to CHT alone.

## Introduction

Cholangiocarcinoma (CC) is a rare tumor with a high mortality, mainly because most patients are diagnosed in locally advanced stages (1). Complete resection of these tumors is the only potentially curative treatment option (2, 3). However, even after achieving curative-intent resection, more than half of the resected patients develop recurrences and long-term outcomes remain discouraging (4–6).

Currently, chemotherapy (CHT) is considered the standard treatment for recurrent cholangiocarcinoma (7, 8). Published studies on surgery for recurrent cholangiocarcinoma are limited. They come mostly from Asian groups, and are characterized by being retrospective and by having, in general, a small number of patients. Furthermore, their results are highly variable, with survival rates ranging from 29% to 100% at 3 years and 0% to 75% at 5 years (9–25). Overall, a surgical approach to CC recurrence seems to be feasible in selected cases; however, since there is a lack of robust studies, the role of surgery is still under debate.

The present study aims to assess the results obtained in our hospital when using surgery for CC recurrences and to evaluate the survival benefit of surgical treatment compared to systemic CHT and best supportive care.

## Methods

We carried out a retrospective study, including all patients who developed recurrence of CC and focused on those who underwent surgical resection at our hospital between January 1995 and December 2015. All patients were followed-up until December 2020 for a minimum of 5 years. Data were obtained from their medical records. The study was performed in accordance with the ethical standards of the Institutional Ethics Committee in our center. Signed informed consent was waived due to the retrospective character of the study and the fact that most of the patients were dead at the time of the study.

The primary outcome was patient survival after diagnosis of recurrent CC. Secondary outcomes included: 1) improvement in survival rate after surgical treatment vs. chemotherapy or supportive care, and 2) morbimortality after surgical treatment.

### Primary tumor

Patients with perihilar cholangiocarcinomas (HiCC) underwent hemihepatectomy or trisectionectomy with en bloc resection of the caudate lobe and extrahepatic bile duct with regional lymph node dissection. For patients with intrahepatic cholangiocarcinoma (ICC), hepatic oncologic resection was applied with the intention to spare the parenchyma. Regional node dissection was not routinely performed. For patients with distal cholangiocarcinoma (DCC), a pancreatoduodenectomy with regional lymph node dissection was performed. After surgical treatment of the primary tumor, all patients were followed by using tumor markers and imaging tests. Adjuvant CHT which comprised Cisplatin-Gemcitabine or 5-Fluorouracil +/- Leucovorin was administered in patients with high-risk factors after resection including affected margins or lymphovascular invasion.

### Recurrence

Recurrence was confirmed by computed tomography (CT) or magnetic resonance imaging (MRI). Positron emission tomography (PET) was also used to evaluate extrahepatic involvement. The first radiological description of recurrence was considered as the date of recurrence. Site of recurrence was categorized according to number as single or multiple and to location as locoregional (when the hepatic hilum, the territory of the hepatic artery, or celiac trunk were involved), hepatic or distant. All patients with CC intraabdominal recurrence were evaluated for a curative resection. Curative-intent surgery for recurrence was considered in patients with technically resectable abdominal recurrences regardless of location. Multiple recurrence was not a contraindication if they were resectable. Patients should show a good performance status (ECOG 0-1). According to our protocol, time to recurrence should exceed 3 months for surgical rescue to be considered. Neoadjuvant chemotherapy was not considered in patients with potentially resectable recurrence. Patients who did not fulfill the criteria for curative-intent surgery were programmed to receive systemic chemotherapy. The best supportive therapy was applied to those with a clinical situation unfavorable to surgery or chemotherapy. All surgical complications were classified using with the Clavien-Dindo scale (26). Adjuvant systemic CHT was indicated in patients who had already undergone surgery based on the same principles used for the primary tumor.

### Statistical analysis

Qualitative variables are summarized as percentages and quantitative variables using the median and interquartile range. Continuous variables were compared in the 3 groups using the Kruskal-Wallis test. Frequencies of characteristics across treatment groups were compared using the Chi-squared test or the Fisher test. Patient and graft survival were analyzed using the Kaplan-Meier method, in which patients lost to follow-up were censored at their last recorded visit. The log-rank test was used to compare survival among the three groups. A univariate Cox regression analysis was performed to identify the patients’ demographic variables, variables related to the primary tumor, or variables related to tumor recurrence, which could be correlated with patient survival. Those variables with a p< 0.10 were included in a multivariate Cox regression model. The proportional hazard assumption was tested. A Microsoft Access database was used and statistical analysis was performed using SPSS 23.0.

## Results

### Characteristics of primary tumors and tumor recurrences

During the study period, a total of 142 patients diagnosed with CC underwent surgery in our hospital, 59 ICC (41.5%), 62 HiCC (43.7%), and 21 DCC (14.8%).

**Figure 1:**
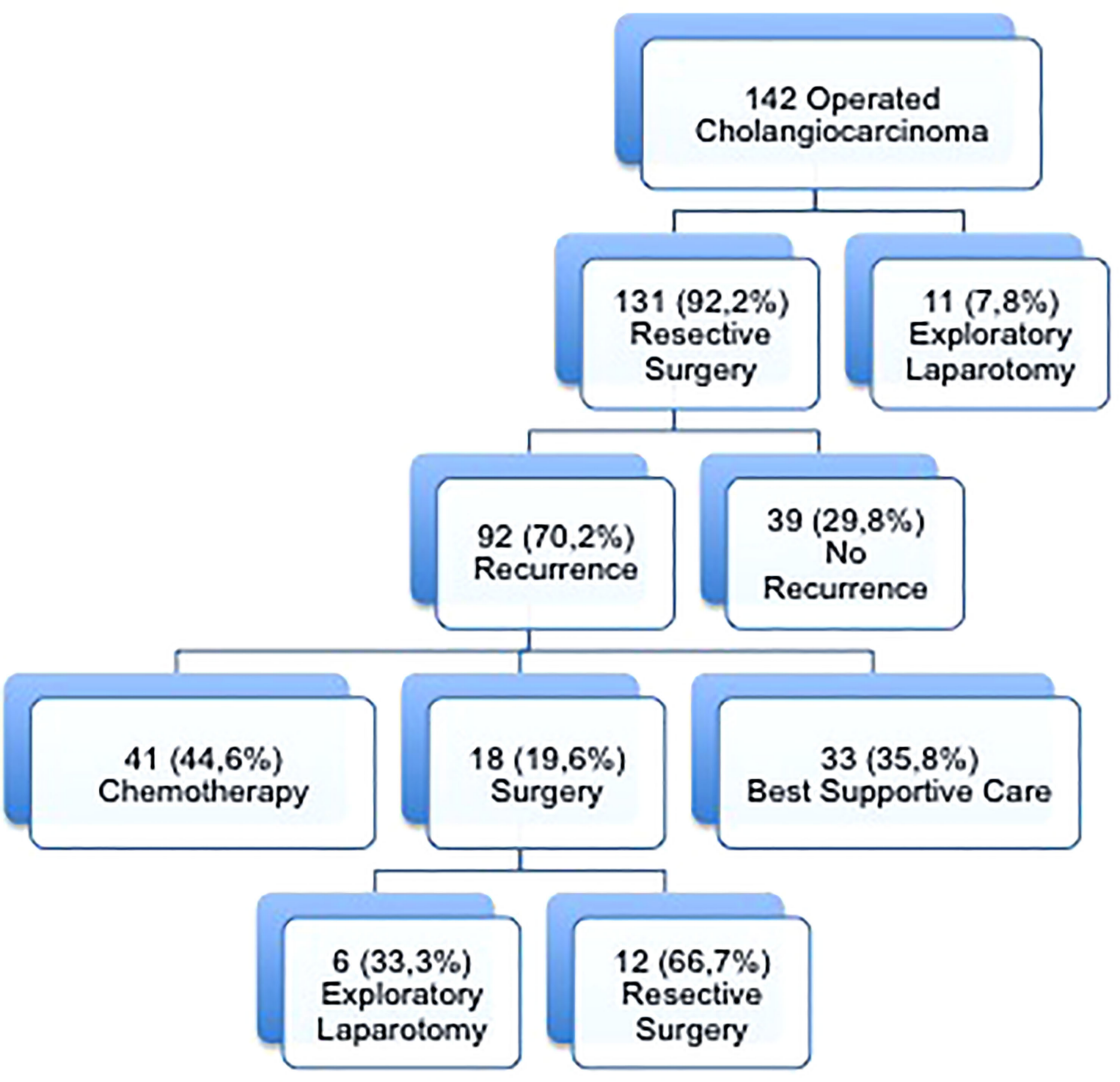
Flowchart of patients with cholangiocarcinoma according to treatment of recurrence.

Of these, 131 patients underwent resection of the primary tumor and 92 (70.2%) developed recurrent disease during the study period. Of these, 41 patients (44.6%) received systemic CHT (the CHT alone group), 18 patients (19.6%) underwent surgery for recurrence and 33 patients (35.8%) received best supportive care. (**Figure 1**).

Patient demographics are shown in **Table 1** according to the treatment received for tumor recurrence.

**Table 1:** Demographics and clinicopathological features of the primary tumor and recurrence according to treatment of the recurrence.

	Total Recurrences N=92	CHT alone N= 41	Surgery N = 18	Supportive Care N = 33	
Primary tumor location					
- **Intrahepatic CC**	34 (37%)	15 (36.6%)	6 (33.3%)	13 (39.4%)	p= 0.49
- **Perihiliar CC**	42 (45,6%)	17 (41.5%)	11 (61.1%)	14 (42.4%)	
- **Distal CC**	16 (17.4%)	9 (21.9%)	1 (5.6%)	6 (18.2%)	
Sex					
- **Male**	57 (62%)	26 (63.4%)	14 (77.8%)	17 (51.5%)	p= 0.209
- **Female**	35 (38%)	15 (36.6%)	4 (22.2%)	16 (48.5%)	
**Age (years)**	64,1 (31-90)	66 (31-76)	63,5 (48-73)	66,5 (41-90)	p= 0.879
ASA					
- **I - II**	43 (46.7%)	21 (51.2%)	7 (38.9%)	15 (45.5%)	p= 0.79
- **III - IV**	49 (53.3%)	20 (48.8%)	11 (61.1%)	18 (54.5%)	
Primary Tumor T					
**- T1 - T2**	61 (66.3%)	26 (63.4%)	14 (77.8%)	21 (63.6%)	p= 0.62
**- T3 - T4**	31 (33.7%)	15 (36.6%)	4 (22.2%)	12 (36.4%)	
Primary Tumor N					
- **N0**	64 (69.6%)	27 (65.8%)	12 (66.7%)	25 (75.8%)	p= 0.79
- **N+**	28 (30.4%)	14 (34.2%)	6 (33.3%)	8 (24.2%)	
Primary Tumor M					
- **M0**	85 (92.4%)	36 (87.8%)	18 (100%)	31 (93.9%)	p= 0.24
- **M+**	7 (7.6%)	5 (12.2%)	0 (0%)	2 (6.1%)	
Primary Tumor resection					
**- R0**	62 (67.4%)	28 (68.3%)	13 (72.2%)	21(63.6%)	p= 0.52
**- R1**	28 (30.4%)	12 (29.3%)	4 (22.2%)	12 (36.4%)	
**- R2**	2 (2.2%)	1 (2.4%)	1 (5.6%)	0 (0%)	
**Adjuvant CHT treatment**	32 (34.8%)	19 (43.2%)	8 (44.4%)	5 (12.2%)	p= 0.003*
Site of Recurrence					
- **Locoregional**	32 (34.8%)	13 (31.7%)	9 (50%)	10 (30.3%)	p= 0.07
- **Hepatic**	32 (34.8%)	18 (43.9%)	4 (22.2%)	10 (30.3%)	
- **Distant**	26 (28.2%)	10 (24.4%)	3 (16.7%)	13 (39.4%)	
- **Multiple**	2 (2.2%)	0 (0%)	2 (11.1%)	0 (0%)	
**Time to recurrence (months)**	11.04 (0-131)	9.9 (0-66)	18.9 (2-82)	11.5 (0-131)	p=0.40

*Best-supportive treatment vs CHT or surgery.

CC, Cholangiocarcinoma; CHT, Chemotherapy.

Data is shown in number (percentage) and median (range).

The most common sites of recurrence were locoregional and the liver both in 34.8% of the patients. Of note, 26,5% of the recurrent ICC showed locoregional recurrence while it was the 45,2% in patients with recurrent HiCC. As for time to recurrence, this was greater than 1 year in 48.4% of patients with a median time of 11 months (range 0-131).

### Characteristics of surgical treatment for recurrences

Detailed patterns of recurrence and surgical procedures are shown in **Table 2**.

**Table 2:** Patients with surgical treatment for cholangiocarcinoma recurrence.

	Location of recurrence	Age	ASA	Primary tumor TNMR	Time to recurrence (months)	Surgery	R status	Surgical complications	Patient survival (months)*
**1**	Locoregional	70	II	HiCC T4N0M0 R0	22	Tumor and RPV en-block resection. RPV reconstruction Hepaticojejnostomy reconstruction	R0	Bile Leakage	9
**2**	Locoregional	57	II	ICC T2N0M0 R0	65	Exploratory laparotomy (Extended retroperitoneal infiltration)	R2	Acute respiratory failure	21
**3**	Locoregional	72	III	HiCC T2N+M0 R0	55	Retrocaval LN and LRV en-block resection	R1		24
**4**	Locoregional	73	III	HiCC T2N+M0R1	7	En-block tumor and PV resection and reconstruction. Hepaticojejunostomy reconstruction	R0	Acute renal failure	1
**5**	Locoregional	52	III	ICC T4N0M0R2	22	Exploratory laparotomy (Celiac trunk infiltration)	R2		42
**6**	Locoregional	48	III	ICC T2N0M0R0	4	Extrahepatic bile duct Hiliar LN. Bile duct resection. Hepaticojejunostomy	R0		24
**7**	Locoregional	73	III	HiCC T1N0M0R0	3	Exploratory laparotomy (Peritoneal carcinomatosis)	R2	SSI	2
**8**	Locoregional	73	II	HiCC T2N0M0R0	8	Exploratory laparotomy (Peritoneal carcinomatosis)	R2		3
**9**	Locoregional	70	II	HiCC T2N+M0R1	10	Exploratory laparotomy (Peritoneal carcinomatosis)	R2		2
**10**	Hepatic	73	III	ICC T1N0M0 R1	82	S-V hepatectomy	R0	Bronchoaspiration; Sepsis; MOD	0
**11**	Hepatic	68	III	ICC T2N0M0R0	6	S-II, III and IVB tumorectomies	R1		39
**12**	Hepatic	55	II	DCC T2N+M0R0	15	S-IVA hepatectomy Partial gastrectomy (Stomach infiltration)	R0		20
**13**	Hepatic	60	III	HiCC T2N0M0R0	13	S-I hepatectomy RFA S-IVA lesion	R0	Intraabdominal collection➔ Percutaneous drainage	50
**14**	Distant	55	II	HiCC T2N0M0 R0	22	Exploratory laparotomy (Extended retroperitoneal infiltration)	R2		31
**15**	Distant	64	III	HiCC T3N+M0R0	17	S-III hepatectomy Hiliar LN	R2	SSI	19
**16**	Distant	63	III	HiCC T3N+M0R1	21	Peritoneal implant resection (Peritoneal implants)	R0		15
**17**	Multiple (Locoregional + Hepatic)	56	II	ICC T2N0M0 R0	21	S-V hepatectomy HA resection and reconstruction Hepatic hilium, celiac trunk, interaortocaval LN	R1	Intraabdominal collection➔ Percutaneous drainage	7
**18**	Multiple (locoregional +hepatic +distant)	56	III	HiCC T2N0M0R0	29	Right anterior liver sectionectomy Hepaticojejunostomy reconstruction Partial gastrectomy	R0	Hemoperitoneum➔ HA bleeding	1

DCC, distal cholangiocarcinoma; HA, hepatic artery; HiCC, hiliar cholangiocarcinoma; IA, intrabdominal; ICC, intrahepatic cholangiocarcinoma; LN, lymphadenectomy; LRV, left renal vein; MOD, multiorgan dysfunction; OS, overall survival; PV, portal vein; RFA, Radiofrequency ablation; RPV, right portal vein; SSI, surgical site infection; TNMR, TNMR stage of TMN classification.

* All patients were dead at the end of the follow-up.

Surgical resection was attempted in 18 patients with CC recurrence. No patient received neoadjuvant chemotherapy. The primary tumor was ICC in 6 patients, HiCC in 11 patients, and DCC in 1 patient. Eleven patients (61.1%) were ASA III-IV. The median time to diagnosis of recurrence was 18.9 months (range 2-82). The recurrence site was locoregional in 61.1% of patients including nine cases of locoregional only and two of multiple recurrence (locoregional and liver), liver only in 22.2%, and distant recurrences in 16.7%.

In six out of the 18 patients, an exploratory laparotomy could only be performed due to unresectable disease. Five of these patients had locoregional recurrence and one distant recurrence. The other 12 patients underwent surgical resection for recurrence (a resectability rate of 66.7%). Surgical resection was possible in four patients with locoregional recurrence only; a new hepaticojejunostomy and portal vein resection with reconstruction were needed in three and two cases, respectively. Four patients with hepatic recurrence were treated with hepatectomy with the addition of partial gastrectomy and radiofrequency ablation in one case each. Two patients with multiple recurrences including locoregional were treated with hepatectomy, hepatic artery resection and lymphadenectomy in one case and hepatectomy, hepaticojejunostomy reconstruction and partial gastrectomy in the other case. As for the resection margin status, R0 was achieved in 8 patients (44.4%).

Regarding morbidity and mortality, 5 patients (27.8%) had severe postoperative complications (≥ grade III on the Dindo-Clavien scale); 3 of these patients died within the first month after surgery (30-day mortality rate was 16.7%).90-day mortality after surgery was 33.3%. Six patients (33.3%) received systemic adjuvant CHT after resection.

### Survival after recurrence treatment

**Figure 2:**
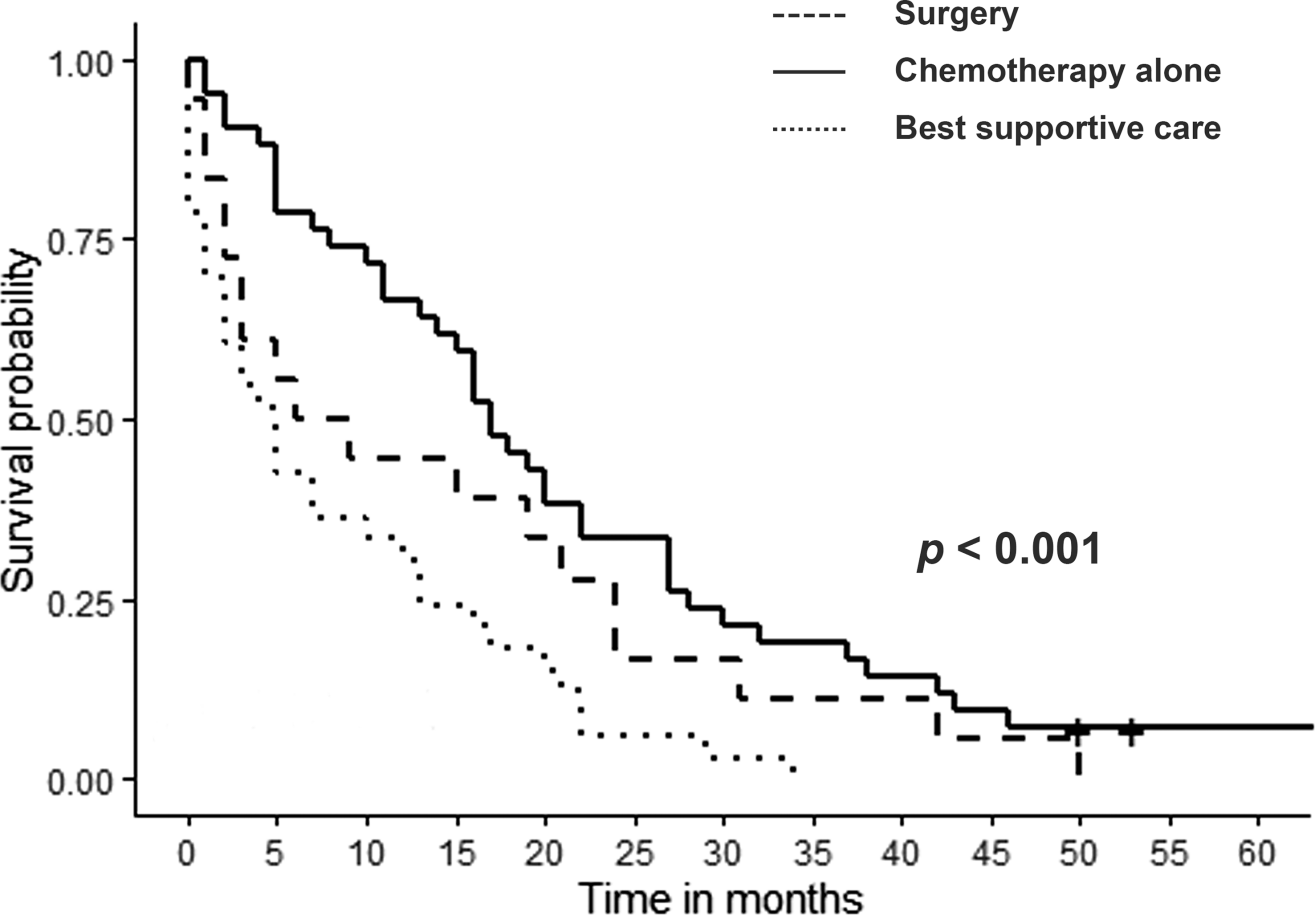
Survival of cholangiocarcinoma recurrence according to the treatment received. Surgery or systemic chemotherapy only vs best supportive therapy (p<0.001). Surgery vs systemic chemotherapy only (p=0.308).

When the three different types of treatment for CC recurrence were compared, no significant differences were found in terms of primary tumor characteristics, site of recurrence or time to recurrence. (**Table 1**). Patients with tumor recurrence were followed up during a median of 13 months (range 0-68,9). The median survival in the groups who underwent surgery, CHT alone and the best supportive care was 15 months (range 0-50), 16.9 months (range 1-69) and 4 months (range 0-34), respectively while 1- and 3-year patient survival rates were 55.6% and 16.6%, 68.3% and 12.2% and 30.3% and 0%, respectively. Survival after any kind of treatment, surgery or CHT alone was significantly better than receiving best supportive care (p< 0.001) (**Figure 2**). Nevertheless, we could not find any significant differences in survival between the patients who received CHT alone and those who underwent surgery (p=0.308).

**Figure 3:**
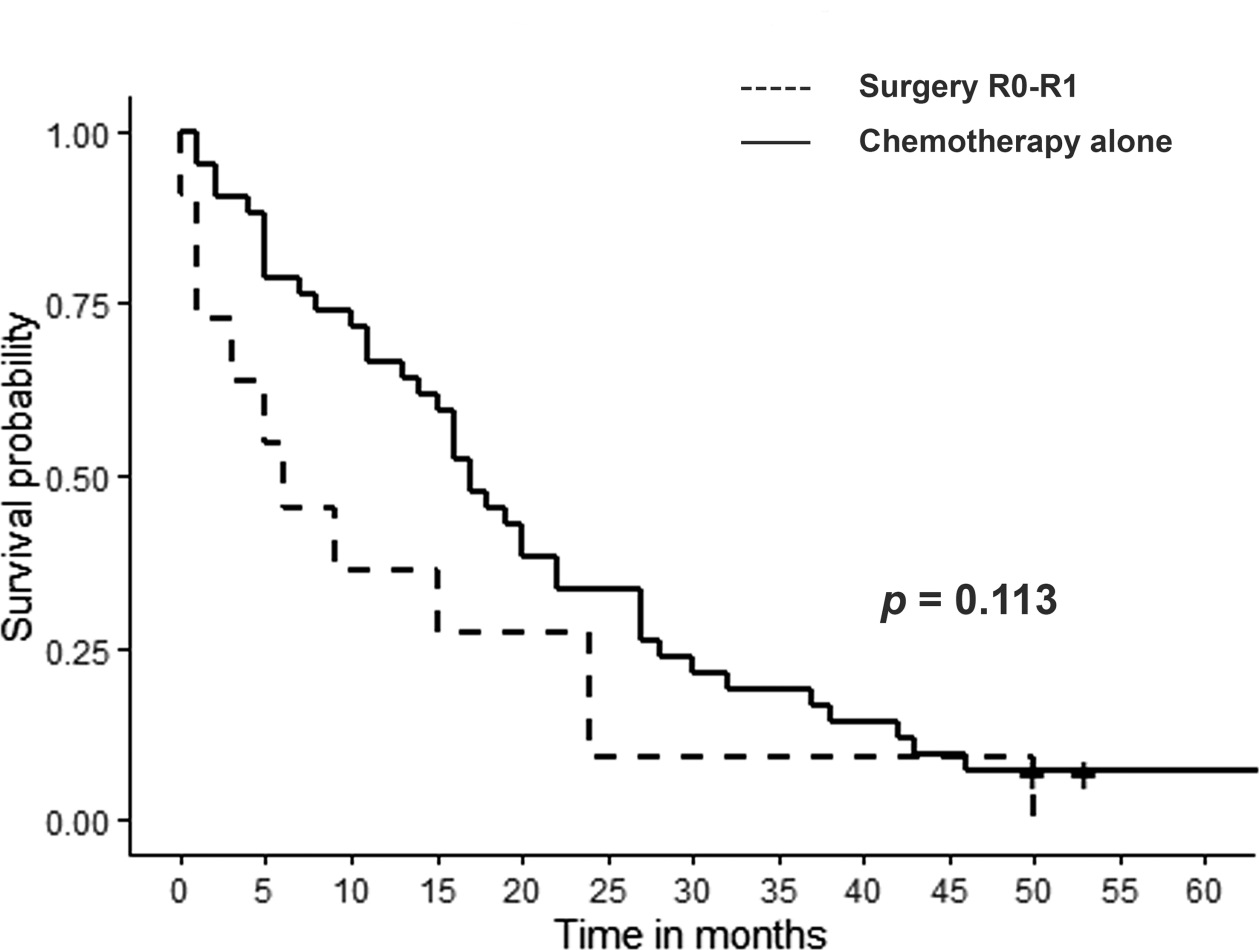
Survival after cholangiocarcinoma recurrence: R0/R1 resection vs systemic chemotherapy only (p=0.113).

The surgical treatment group was analyzed in greater depth. The median survival time of 11 patients who underwent surgery with curative intent (R0/R1), was 15 months (range 0.9-50) with a 1- and 3-year patient survival rate of 54.5% and 18.1%, respectively. This survival rate was not better than the survival rate of the patients who received CHT alone (p= 0.113) (**Figure 3**). Finally, we analyzed the most favorable patients, those who received surgery with curative intent (R0) and did not die after surgery (n=5). The median survival of these patients was 23.9 months (range 4.7-50) with a 1- and 3-year patient survival rate of 80% and 20%, respectively which, again, was not better than the survival rate obtained with CHT alone (p= 0.928). Of note, only three patients in the surgery group survived more than 3 years after the procedure though all were death at the end of the study. One patient died after developing a new cholangiocarcinoma recurrence and two patients died because of non-oncological reasons.

### Univariate and multivariate analysis

In the univariate analysis, only a time to recurrence of less than 1 year was independently related to mortality after diagnosis of CC recurrence (HR = 1.76 CI 95% [1.13-2.74], p=0.013) (**Table 3**). Furthermore, systemic adjuvant CHT after surgery for the primary tumor was a significant protective factor (HR = 0.51 CI 95% [0.31-0.82], p=0.006), as was having received any treatment, CHT alone or surgical treatment, for recurrence instead of supportive care (HR = 0.35 CI 95%. [0.21-0.58], p=<0.001).

**Table 3:** Univariate and multivariate analysis of risk factors for mortality after cholangiocarcinoma recurrence.

	Univariate		Multivariate	
	p	HR (IC 95%)	p	HR (IC 95%)
**Sex (Female)**	0.884	1.03(0.66-1.62)		
**Age (>= 65 years)**	0.716	1.08(0.69-1.68)		
**T (T3-4)**	0.795	1.06(0.67-1.69)		
**N +**	0.675	1.11(0.69-1.77)		
**M +**	0.219	1.63(0.74-3.57)		
**R status (ref R0)** - **R1** - **R2**	0.542 0.419 0.512	1.23(0.75-2.01) 0,62(0.15-2.59)		
**Adjuvant CHT (yes)**	0.006	0.51(0.31-0.82)	0.013	0.52(0.31-0.87)
**Time to recurrence < 1 year**	0.013	1.76(1.13-2.74)	0.001	2.22(1.39-3.54)
**Recurrence Treatment:** **CHT or Surgery VS Supportive Care**	<0.001	0.35(0.21-0.58)	<0.001	0.34(0.20-0.58)

CHT, Chemotherapy.

In the multivariate analysis, these 3 factors remained as the only significant prognostic factors related to mortality after recurrence (**Table 3**).

## Discussion

The literature addressing surgical treatment for recurrent CC is scarce and is limited to short series (10 out of 14 series with fewer than 30 patients). Most studies focus on intrahepatic tumors (14–22), while others include all kinds of CC regardless of their location (9–12, 23). The published studies on surgery for recurrent CC are listed in **Table 4**. Overall, studies focused on ICC recurrences show that surgical treatment is more frequently indicated and achieves better survival outcomes in these cases when compared with those studies that include all kinds of CC. This can be explained by the fact that perihiliar CC more often presents locoregional unresectable recurrences. Si et al. published the most extensive study with 72 patients with recurrent ICC and reported survival rates at 3 and 5 years of 53% and 35.3%, respectively (21). Nevertheless, the few studies that analyze results after surgery for recurrent CC regardless of the primary tumor’s location report median survival times of between 18.9 and 28.5 months and survival rates at 3 and 5 years of 31%-38% and 14%-23% respectively (9–12). Therefore, the actual benefit of the surgical treatment on survival compared to CHT alone cannot be determined accurately (10).

**Table 4:** Published studies of the surgical treatment for cholangiocarcinoma recurrence.

Author	Year	N	Type of Cholangiocarcinoma	Median survival after surgical treatment (months)	3- year survival after surgical treatment (%)	5- year survival after surgical treatment (%)
**Yamamoto** (14)	2001	4	ICC	10	29	29
**Ohtsuka** (15)	2009	9	ICC	22	51.4	51.4
**Kamphues** (17)	2010	9	ICC	51	52	34.4
**Ercolani** (16)^*^	2010	6	ICC	66.6	60	0
**Saiura** (18)	2011	5	ICC	Not available	80	60
**Sulpice** (19)	2012	4	ICC	Not available	100	75
**Spolverato** (20)	2016	41	ICC	26.1	Not available	Not available
**Si** (21)	2017	72	ICC	25.6	53	35.3
**Bartsch** (22)	2019	17	ICC	65.2	85	62
**Song** (9)	2011	27	ICC + HiCC + DCC	18.9	Not available	Not available
**Takahashi** (10)	2015	74	Biliary tract**	Not available	37	14
**Noji** (11)	2015	27	Biliary tract excluding ICC	Not available	31	23
**Miyazaki** (12)	2017	14	Biliary tract**	28.5	38	19
**Sakata** (23)	2021	52	Biliary tract**	33	50	29
**Current Study**	2021	18	ICC + HiCC + DCC	15	16.6	0

* Includes all treated patients (surgery or radiofrequency ablation).

** Biliary tract includes Gall bladder.

ICC, Intrahepatic cholangiocarcinoma; HiCC, Hiliar cholangiocarcinoma; DCC, Distal cholangiocarcinoma.

Recently, Sakata et al. have reported the outcomes of surgery in recurrent biliary tract cancer in a single-center series with 24 patients and a multicenter series with 54 patients (23). Recurrence was single distant in 46% and locoregional in 26.9% of cases. 3- and 5-year cancer specific survival rates after surgery for recurrence were 50% and 29%, respectively. Of note, the only independent factor associated with survival was the initial site of recurrence with single-distant recurrence being the most favorable.

In our study, recurrences of all kinds of CC were included although gallbladder cancer was excluded. Primary tumor features were comparable across the three treatment groups: surgery, CHT alone, or best supportive care. We found a survival benefit in patients who received any kind of treatment, CHT alone or surgery, as compared to those who only received best supportive care. Nevertheless, we could not demonstrate any survival benefit in patients who underwent surgery versus those who received CHT alone. The survival rate at 1 and 3 years after surgery was 55.6% and 16.6%, respectively. Indeed, postoperative mortality had a significantly negative effect on survival in our study. Our rate of major complications (27.8%) and 30-day mortality (16.7%) were high when compared with the literature. These data reflect the surgical challenge that CC recurrence may pose. Our grim results can be explained by the combination of the clinical status of our patients and the aggressiveness of the surgical procedures in relationship with the high incidence of locoregional recurrence. In our experience, 61.1% of the surgical patients were ASA III and locoregional recurrence was observed in the same proportion. On the one hand, 45,5% of the patients with locoregional recurrence underwent exploratory laparotomies. On the other hand, 12 patients finally underwent challenging resections of the CC recurrence, seven (58.3%) were treated with different types of hepatectomies, four (33.3%) needed reconstruction of the hepaticojejunostomy, three (25%) underwent vascular resection with reconstruction and two (16.6%) partial gastrectomy. Overall, 66.6% of the patients received some combination of aggressive surgical techniques. These surgical techniques clearly show our aggressiveness in the search for a curative surgical treatment. Nevertheless, despite of our aggressive attitude, less than 20% of the patients with CC recurrence were operated and only 13% were finally resected.

A good selection of the patients to be treated with surgery seems to be the key to improving outcomes after CC recurrence. A good balance between the patients’ medical status and the aggressiveness of the planned surgery may reduce postoperative morbidity and mortality. Nevertheless, accurate presurgical evaluation is not easy in patients with recurrent CC as our experience shows with a 33.3% rate of exploratory laparotomies and a 44% rate of R0 surgeries versus 92.3% and 68.7%, respectively after surgical treatment of the primary tumor. This is even more evident for locoregional recurrence. In these patients, the rate of exploratory laparotomies increased to 45.5% and R0 surgeries decreased to 36.4% in our experience. In line with this, recent experiences have shown that the location of recurrence is an independent factor in survival after surgical treatment with single-distant recurrence yielding the best results (23). In any case, it is noteworthy that in our study, none of the surgically treated patients, even those with a R0 resection, were alive after 5 years.

In our study, multivariate analysis identified surgical treatment or CHT alone, time to recurrence greater than 1 year, and adjuvant CHT after the primary tumor surgery as significant prognostic factors for mortality after CC recurrence. Time to recurrence greater than 1 year as an independent prognostic factor for survival in most of the studies (10, 12). In addition, adjuvant CHT has shown survival benefits in patients with high-risk features after surgery (25, 27–29). In this sense, the BILCAP study showed survival benefits of adyuvant CHT with capecitabine in patients with biliary tract cancer resected with curative intent (30). Our study seems to be the first to identify adjuvant CHT after primary tumor surgery as an independent prognostic factor for survival after CC recurrence (9, 10, 12).

Systemic CHT with gemcitabine and cisplatin is the current standard of care for metastatic and locally advanced biliary cancer as well as for those patients with recurrence (25, 31). Our experience is consistent with this idea and does not favour surgery for the treatment of CC recurrence. However, it is still feasible that curative-intent surgery could play a role in highly selected patients, provided that less aggressive surgery is needed.

We recognize limitations to our study. It is retrospective with a limited number of patients, which prevents us from analyzing the behavior of different types of CC independently. A possible selection bias in the selection of patients for surgical treatment may have occur during the study period, although the surgeons involved in the decisions to operate did not change over that time. We must also admit that diagnosis, staging and overall management of CC may have evolved during the period of our study; however, the real evolution has occurred after the end of our study with the development of new diagnostic tools allowing 3D reconstructions and more accurate decisions regarding resectability, new systemic therapies and different surgical attitudes including regional lymphadenectomy in ICC (25, 27, 32). Nevertheless, treatment of CC recurrence has not evolved significantly in the last decade and systemic chemotherapy is still the preferred approach for patients with locally recurrent or metastatic disease (25).

Finally, neoadjuvant chemotherapy was not considered before surgical treatment and this might have influenced the outcomes according to the current knowledge. Nevertheless, this was a common practice at the time of the study as reported by other authors (21, 23).

Considering the scarcity of published studies, the novelty of our report lies in the fact that it represents one of the few western experiences to date and shows a different vision of the role of surgery for the treatment of recurrent CC due to a high rate of exploratory procedures and a high postoperative mortality mainly related to the aggressiveness of the surgical approach in patients with locoregional recurrence. We believe that our study can be of help to properly assess the role of surgery in CC recurrence.

In summary, in our experience, surgery for CC recurrence was limited to a small group of patients. Although feasible, it was technically demanding, and unfortunately, was characterized by frequent exploration procedures and a high postoperative mortality rate that affected survival outcomes. We found that any therapeutic action such as surgery or CHT alone was better than best supportive care in terms of patient survival; however, in our hands, no significant survival benefit for surgery was demonstrated over CHT alone, even in those patients in whom curative resection was accomplished. Nevertheless, according to literature some selected patients mainly based on the location of recurrence and the aggressiveness of the needed surgery might benefit from a curative-intent surgery. This might include single-distant or hepatic recurrence requiring limited surgical procedures. Since worldwide experience is limited to small retrospective cohorts and results after surgery are variable, an international registry focused on the surgical treatment of the CC recurrence would be desirable.

## Data availability statement

The raw data supporting the conclusions of this article will be made available by the authors, without undue reservation.

## Ethics statement

The studies involving human participants were reviewed and approved by Cruces University Hospital's ethics committee. Signed informed consent was waived due to the retrospective character of the study and the fact that most of the patients were dead at the time of the study.

## Author contributions

Material preparation, data collection and analysis were performed by LF, MG, EA and MP. The first draft of the manuscript was written by LF and all authors commented on previous versions of the manuscript. All authors contributed to the article and approved the submitted version. This manuscript has not been published before and is not under consideration for publication elsewhere.
